# *In Silico* Virome Analysis of Chinese Narcissus Transcriptomes Reveals Diverse Virus Species and Genetic Diversity at Different Flower Development Stages

**DOI:** 10.3390/biology12081094

**Published:** 2023-08-05

**Authors:** Hoseong Choi, Yeonhwa Jo, Won Kyong Cho

**Affiliations:** 1 Plant Health Center, Seoul National University, Seoul 08826, Republic of Korea; bioplanths@gmail.com; 2 College of Biotechnology and Bioengineering, Sungkyunkwan University, Suwon 16419, Republic of Korea; yeonhwajo@gmail.com

**Keywords:** Chinese narcissus, virome, transcriptome, viral contigs, viral genomes, phylogenetic analysis, genetic diversity

## Abstract

**Simple Summary:**

In this study, we analyzed Chinese narcissus flowers at different stages of development to determine the presence of viruses. We identified seven viral species, including narcissus common latent virus (NCLV), narcissus mottling-associated virus (NMaV), and narcissus symptomless virus (NSV) from the genus *Carlavirus*. We also found contigs associated with cucumber mosaic virus (CMV) and three viruses from the genus *Potyvirus*: narcissus degeneration virus (NDV), narcissus late season yellow virus (NLSYV), and narcissus yellow stripe virus (NYSV). NCLV was the most abundant among these viruses, followed by NYSV and NMaV. The composition of viruses varied between white tepal plants and yellow tepal plants. White tepal plants harbored six viruses, whereas yellow tepal plants harbored seven viruses, including CMV. The number of viral reads increased as flowers developed in white tepal plants; however, the difference in yellow tepal plants was not statistically significant. NDV was the most common virus in the white tepal samples, whereas NDV and NYSV were prominent in the yellow tepal samples. The viruses showed distinct genetic groups based on phylogenetic analysis, and genetic diversity analysis revealed variations in sequence similarities and differences within each virus. This study provides valuable insights into the identification, abundance, composition, and genetic diversity of Chinese narcissus flowers at different developmental stages.

**Abstract:**

Viromes of Chinese narcissus flowers were explored using transcriptome data from 20 samples collected at different flower development stages. Quality controlled raw data underwent *de novo* assembly, resulting in 5893 viral contigs that matched the seven virus species. The most abundant viruses were narcissus common latent virus (NCLV), narcissus yellow stripe virus (NYSV), and narcissus mottling-associated virus (NMaV). As flower development stages advanced, white tepal plants showed an increase in the proportion of viral reads, while the variation in viral proportion among yellow tepal plants was relatively small. Narcissus degeneration virus (NDV) dominated the white tepal samples, whereas NDV and NYSV prevailed in the yellow tepal samples. Potyviruses, particularly NDV, are the primary infectious viruses. *De novo* assembly generated viral contigs for five viruses, yielding complete genomes for NCLV, NDV, narcissus late season yellow virus (NLSYV), and NYSV. Phylogenetic analysis revealed genetic diversity, with distinct NCLV, NMaV, NDV, NLSYV, and NYSV groups. This study provides valuable insights into the viromes and genetic diversity of viruses in Chinese narcissus flowers.

## 1. Introduction

Narcissus, a genus of flowering plants belonging to Amaryllidaceae, encompasses a diverse group of ornamental flowers renowned for their beauty and fragrance [1]. Chinese narcissus, also known as Chinese daffodil (*Narcissus tazetta*), is a widely cultivated ornamental flower known for its delicate allure [2]. It is culturally and economically important as an ornamental plant in China and other parts of the world. However, like many other plant species, this plant is susceptible to viral infections that can severely affect its growth, development, and aesthetic value [3]. Viral diseases affecting *Narcissus tazetta* can reduce flower quality, hinder growth, and cause significant economic losses to the horticultural industry [4].

Narcissus is primarily affected by various plant viruses, including members of families such as *Potyviridae*, *Alphaflexiviridae*, and *Carlaviridae* [3,5,6,7,8]. The most common viruses infecting the *Narcissus* species from the *Potyviridae* family include narcissus late season yellows virus (NLSYV) [9], narcissus yellow stripe virus (NYSV) [5], narcissus degeneration virus (NDV) [6], and narcissus latent virus (NLV). Additionally, narcissus common latent virus (NCLV) and narcissus symptomless virus (NSV) in the *Carlaviridae* family have been identified [7]. Several previous studies have demonstrated that infection of narcissus plants by multiple viruses is widespread [3,4,10,11]. These viruses can be transmitted through various means, including insect vectors, contaminated tools, and vegetative propagation. When the narcissus is infected with viruses, viral pathogens can persist and spread, resulting in a decline in flower quality, stunted growth, leaf yellowing, and plant death.

The study of plant viromes, encompassing the total viral population within a plant, is crucial for understanding the diversity and dynamics of plant-associated viruses [12,13]. Advanced molecular techniques, such as high-throughput sequencing (HTS), have revolutionized the study of plant viromes by enabling comprehensive analyses of viral populations present within host organisms [14,15]. In particular, RNA sequencing (RNA-seq) has emerged as a powerful tool for virome analysis, allowing for the identification and characterization of viral sequences in plant samples [16,17]. By leveraging RNA-seq data, researchers can gain insights into viral species, their genetic diversity, and their interactions with the narcissus host [18]. Understanding viral infection diversity, prevalence, and the impact on narcissus is crucial for developing effective disease management strategies.

This study aims to investigate the viromes associated with Chinese narcissus flowers at different flowering stages using RNA-seq data. By capturing the viral genetic material in the flower samples, we can understand the viral diversity and dynamics throughout the flowering process. Additionally, by examining viral populations at different flowering stages, we can explore potential correlations between viral abundance and the developmental stages of the flowers.

## 2. Materials and Methods

### 2.1. Sample Collection and RNA-Seq Library Preparation

In this study, we utilized transcriptome data from a previous study that performed RNA-seq analysis on two *Narcissus tazetta* cultivars, namely ‘Jinzhan Yintai’ with white tepal and its yellow tepal mutant [19]. Total RNA was extracted from the collected tissues, followed by cDNA library preparation using oligo dT for RNA-seq, as previously described [19]. A total of 20 paired-end libraries (150 bp × 2) were sequenced using the Illumina NovaSeq 6000 system.

### 2.2. Raw Data Processing and De Novo Transcriptome Assembly

The raw datasets (SRR19993640-SRR19993659) associated with accession number PRJNA855612 were obtained from the Sequence Read Archive (SRA) database at the National Center for Biotechnology Information (NCBI). The downloaded SRA data were converted into the FASTQ format using the SRA-Toolkit 3.0.5 (https://hpc.nih.gov/apps/sratoolkit.html). All software and data mentioned in the Section 2 were accessed on 1 June 2023. The commands (codes) for the programs utilized in the Section 2 are available in the Supplementary Methods. Raw FASTQ files were subjected to quality control, including trimming and filtering of low-quality reads, using BBDuk 39.01 (https://jgi.doe.gov/data-and-tools/software-tools/bbtools/bb-tools-user-guide/bbduk-guide/). The resulting high-quality reads were subjected to *de novo* transcriptome assembly using Trinity version 2.15.0 with default parameters [20].

### 2.3. Identification of Viral Contigs by BLASTX Search

The contigs assembled from each library were subjected to a BLASTX search against the viral protein database derived from the NCBI for Biotechnology Information (https://www.ncbi.nlm.nih.gov/genome/viruses/), using an E-value cutoff of 1E-10. Contigs showing sequence similarity to viral proteins were further subjected to a BLASTX search against the non-redundant protein database at NCBI to identify the viral contigs specifically. Identified viral contigs were categorized based on their corresponding viral species. To align the raw sequence reads against the reference viral genomes, we used BWA aligner version 0.7.17 with default parameters [21]. The coverage, viral reads, and transcripts per million (TPM) for each virus in individual samples were calculated using the eXpress 1.5.1 program (https://pachterlab.github.io/eXpress/manual.html) based on the SAM file.

### 2.4. Annotation of the Viral Genome

Only viral contigs covering the complete open reading frames (ORFs) were used to annotate the viral genome using the ORFfinder program (https://www.ncbi.nlm.nih.gov/orffinder/) available at NCBI. The reverse sequences were converted using a DNA Reverse Complement Calculator (https://jamiemcgowan.ie/bioinf/complement.html). The predicted ORFs were subjected to a BLASTX search against a non-redundant protein database to identify viral genomic features, such as conserved motifs and potential functional elements, by comparing them with previously known viral genomes.

### 2.5. Phylogenetic Analysis

We downloaded all available viral genome sequences from the NCBI GenBank database to construct the phylogenetic trees. Viral sequences were aligned using MAFFT version 7 with the auto option [22]. The aligned sequences were trimmed by removing the 5′ and 3′-UTRs. Subsequently, the aligned sequences were substituted for model determination, followed by phylogenetic tree construction using IQ-TREE version 1.6.12, with the maximum likelihood method and 1000 bootstrap replicates [23]. The resulting phylogenetic trees were visualized and edited using Figtree version 1.4.4 (http://tree.bio.ed.ac.uk/software/figtree/).

### 2.6. Genetic Diversity Analysis

The aligned sequences used for phylogenetic tree construction were used for genetic diversity analysis using DnaSP6 version 6.12.03 [24]. The genetic diversity indices calculated included the total number of sites, number of segregating sites (S), total number of mutations (Eta), number of haplotypes (H), haplotype diversity (Hd), nucleotide diversity (Pi), and Watterson’s estimator of θ (θw).

### 2.7. Recombination Analysis

Recombination analysis was performed using RDP version 5.23 [25]. A comprehensive exploratory recombination scan incorporated seven detection methods (RDP, GENECONV, Bootscan, Maxchi, Chimaera, SiScan, and 3Seq). Recombinants predicted by at least four algorithms with a *p*-value less than 0.05 were considered true recombinants. We cross-validated the identified recombination events using all seven detection methods.

## 3. Results

### 3.1. Identification of Viruses from Chinese Narcissus Transcriptomes

To investigate the viromes of narcissus flowers, we utilized Chinese narcissus transcriptomes comprising 20 distinct samples obtained from a previous study [19]. Samples were collected at five different stages of flower development: the bud stage (S1), initial flowering stage (S2), full bloom stage (S3), full expansion stage (S4), and decay stage (S5), from two Chinese narcissus cultivars (Table 1). Two independent biological replicates were used at each stage. To facilitate sample identification, we assigned the names W (white) and Y (yellow) to the plants based on the color of their tepals. For instance, W1R1 indicates White, Stage 1, Repetition 1. Each developmental stage is represented by numerical values ranging from 1 (S1) to 5 (S5). The biological replicates within each stage were classified as R1 (replicate 1) and R2 (replicate 2).

**Figure 1:**
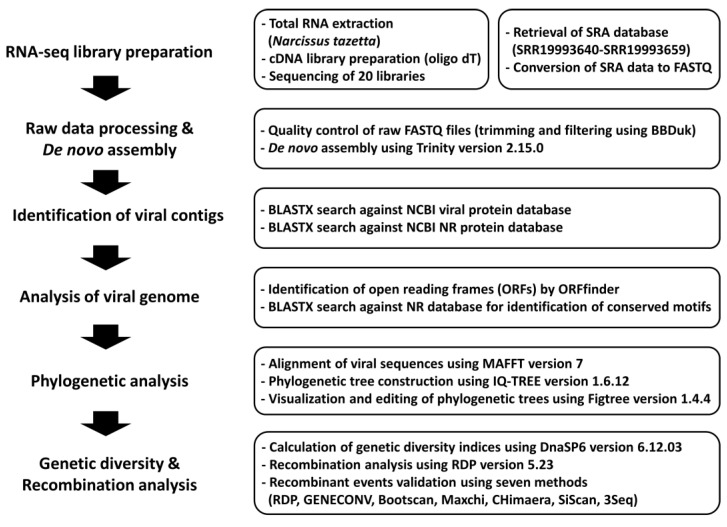
Schematic representation of the narcissus virome data analysis workflow using narcissus transcriptome data.

As depicted in Figure 1, the viromes of narcissus were analyzed using data from 20 narcissus transcriptomes. Initially, raw data for each sample were retrieved from the NCBI SRA database to identify viruses present within the 20 Chinese narcissus flower transcriptomes. Subsequently, quality control measures were applied, and trimming was performed to ensure data integrity. The raw data within each library were subjected to *de novo* assembly using Trinity assembler. The resulting assembled contigs were compared to a viral protein database using BLASTX. Contigs identified as contaminants originating from narcissus were removed from further analysis. Ultimately, we obtained 5893 viral contigs that matched seven distinct viral species.

Within the genus *Carlavirus*, we identified three viruses: NCLV (2913 contigs), narcissus mottling-associated virus (NMaV) (463 contigs), and NSV (30 contigs). In addition, six contigs were associated with the cucumber mosaic virus (CMV) genome, including RNA1 (three contigs), RNA2 (one contig), and RNA3 (two contigs). Furthermore, we identified three viruses within the genus *Potyvirus*: NDV (266 contigs), NLSYV (360 contigs), and NYSV (1855 contigs). Notably, NCLV (2913 contigs) demonstrated the highest abundance among the identified viruses, followed by NYSV (1855 contigs) and NMaV (463 contigs), as determined by the number of contigs.

Using viral sequences as references for the seven identified viruses, we performed read mapping of the raw data from each library to calculate the number of mapped reads and transcripts per million (TPM) for each virus (Table 2). It should be noted that, for NMaV, only a partial sequence covering the viral replicase was available for analysis. Eighteen million, three hundred and seventy two thousand, three hundred and thirty one reads were successfully mapped to seven viral genomes (Table 2) and based on the highest read count NYSV (40.6%) was followed by NDV (32.3%), NCLV (12.4%), and NMaV (10.7%).

### 3.2. Number of Identified Viruses and Proportion of Viral Reads in Each Sample

Six viruses were identified in the white tepal plants, whereas seven were detected in the yellow tepal plants. Specifically, the composition of the identified viruses in the white tepal samples remained consistent across all samples. In contrast, two samples, Y4R1 and Y4R2, were infected with all seven viruses, including CMV, whereas the remaining eight yellow tepal samples were infected with six viruses.

**Figure 2:**
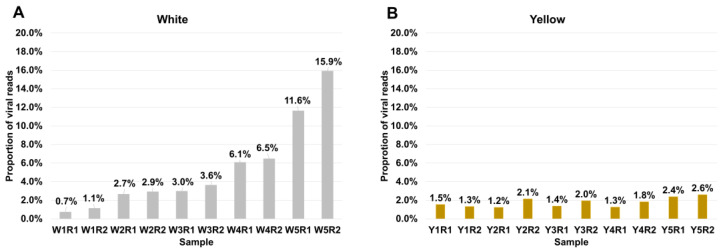
Number of identified viruses and viral proportion in each sample. (**A**) The proportion of viral reads in white tepal plants. (**B**) The proportion of viral reads in yellow tepal plants. The proportion of viral reads was calculated by dividing the total viral reads by the total reads in each library. The graph was generated using Microsoft Excel 2021.

Subsequently, we assessed each sample’s proportion of viral reads (Figure 2). Notably, in the white tepal samples, the proportion of viral reads gradually increased with the advancement of flower development (Figure 2A). The viral proportions in W1R1 and W1R2 were 0.7% and 1.1%, respectively. However, the viral proportion increased significantly to 11.6% in W5R1 and 15.9% in W5R2. In contrast, the difference in the viral proportions among the yellow tepal samples was relatively small, ranging from 1.2% to 2.6% (Figure 2B). When comparing the viral proportion at flower development stage 1, such as Y1R1 (1.5%) and Y1R2 (1.3%), with that at stage 5, such as Y5R1 (2.4%) and Y5R2 (2.6%), a slight increase in the viral proportion was observed (Figure 2B).

### 3.3. Number of Identified Viruses and Proportion of Viral Reads in Each Sample

**Figure 3:**
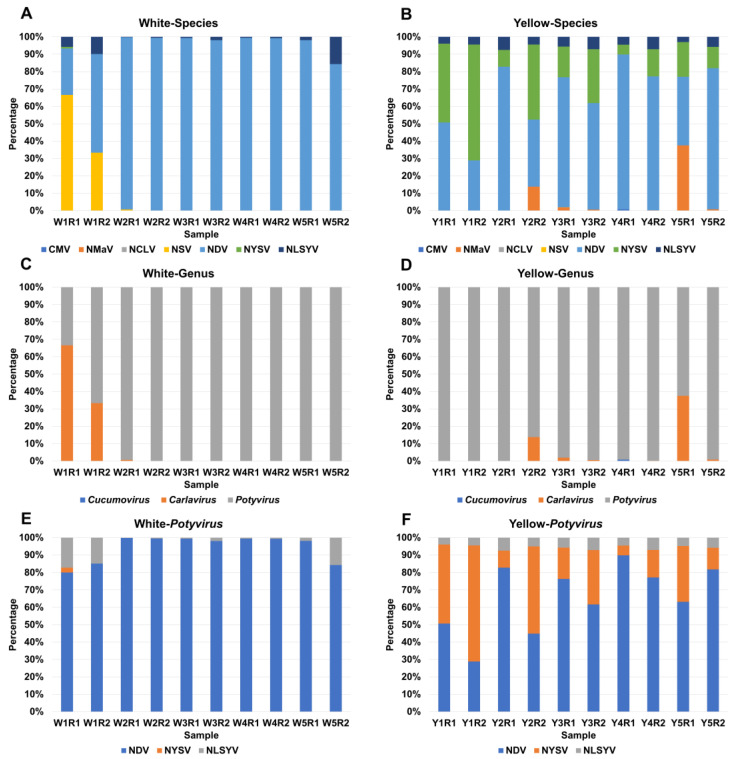
Composition of identified viruses in each sample based on TPM value. (**A**) Percentage of identified viral species in each sample for white tepal plants. (**B**) Percentage of identified viral species in each sample for yellow tepal plants. (**C**) Percentage of identified viral genus in each sample for white tepal plants. (**D**) Percentage of identified viral genus in each sample for yellow tepal plants. (**E**) Percentage of identified potyviruses in each sample for white tepal plants. (**F**) Percentage of identified potyviruses in each sample for yellow tepal plants. We utilized the transcripts per million (TPM) values for each virus to unveil the composition of identified viruses in each sample. First, we combined the TPM values for all the viruses identified in a particular sample to obtain a total TPM value. With the combined total TPM values in hand, we calculated the percentage of each virus based on its species, genus, and family representation. This approach allowed us to understand the relative abundance and distribution of different viruses within each sample.

Examining the composition of the identified viruses in each sample can provide valuable insights into the dominant viruses in samples infected with multiple viruses. Among the ten white tepal samples, NDV emerged as the most abundant virus in nine samples, ranging from 56.7% (W1R2) to 99.4% (W2R2 and W4R1), except W1R1, where NSV (66.6%) was the dominant virus, followed by NDV (26.7%) (Figure 3A). Furthermore, the proportion of NLSYV exceeded 5% in three samples: W1R1 (5.7%), W1R2 (9.9%), and W5R2 (15.6%) (Figure 3A).

In the yellow tepal samples, NDV emerged as the dominant virus in eight samples, ranging from 39.5% (Y5R1) to 89% (Y4R1) (Figure 3B). NYSV was also prominent in most of the yellow tepal samples, ranging from 5.6% (Y4R1) to 66.6% (Y1R2) (Figure 3B).

Except for two samples, W1R1 and W1R2 contained several carlaviruses (Figure 3C). In yellow tepal samples, potyviruses were dominant in eight samples, except for Y2R2 and Y5R1, which also harbored carlaviruses (Figure 3D).

In this study, potyviruses appeared to be the major viruses infecting *Narcissus tazetta*. Next, we analyzed the proportion of identified potyviruses in each sample to identify the dominant species. In the white tepal samples, NDV emerged as the dominant potyvirus in most samples, with NLSYV also being abundantly present in W1R1, W1R2, and W5R2 (Figure 3E). In the yellow tepal samples, NDV was the most abundant potyvirus among the seven samples, followed by NYSV, which was prominently present in Y1R2 and Y2R2 (Figure 3F). NLSYV was identified in all yellow tepal samples, but its proportion ranked third among the three potyviruses infecting Chinese narcissus (Figure 3F).

### 3.4. Assembly and Annotation of Viral Genomes

Through *de novo* transcriptome assembly, we obtained numerous contigs from five different viruses (Table 3). The size of the acquired viral contigs ranged from 201 bp to 1000 bp. Specifically, most viral contigs associated with NMaV, for which the genome sequence was unavailable, were less than 1000 bp. Only six viral contigs associated with NMaV had sizes between 1001 bp and 2000 bp. The number of viral contigs linked to CMV and NSV was limited, and these contigs were shorter than those in the reference viral genomes.

The reference genome of NCLV (GenBank NC_008266.1) was 8539 nucleotides (nt). Among the 24 viral contigs associated with NCLV, more than 8001 base pairs (bp) were observed. The genome sizes of three potyviruses, NDV (GenBank NC_008824.1) with 9816 nt, NLSYV (GenBank NC_023628.1) with 9687 nt, and NYSV (GenBank NC_011541.1) with 9650 nt, exceeded 9000 nt. It is plausible to consider 41 viral contigs (NYSV), 5 viral contigs (NLSYV), and 27 viral contigs (NDV) as putative viral genome sequences using a cutoff of 9000 bp.

Candidate viral contigs for the identified viruses were further analyzed to identify open reading frames (ORFs). Only viral genomes encompassing complete open reading frames (ORFs) were selected. We obtained 18 NCLV, 27 NDV genomes, 2 NLSYV genomes, and 33 NYSV genomes (Table S1).

### 3.5. Phylogenetic Analysis of Identified Viral Genomes

Phylogenetic trees were constructed for the five viruses based on the obtained viral genomes and known reference sequences from GenBank. For NCLV, NDV, NLSYV, and NYSV, only nucleotide sequences encompassing the complete open reading frames (ORFs) were used for tree construction.

**Figure 4:**
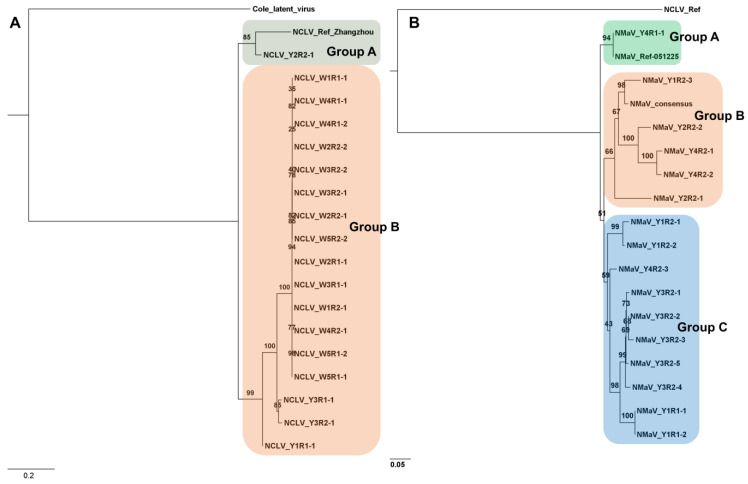
Phylogenetic trees of NCLV isolates and NMaV isolates. (**A**) A phylogenetic tree was constructed using the complete ORFs of 19 NCLV isolates. The maximum likelihood method with 1000 bootstrap replicates was used, and NCLV (GenBank NC_008266.1) and cole latent virus (GenBank MK770418.1) were used as the reference genome and outgroup, respectively. The best-fit model for Bayesian Information Criterion (BIC) was SYM + I + G4. (**B**) Phylogenetic tree constructed using partial sequences of 18 NMaV isolates. The maximum likelihood method with 1000 bootstrap replicates was used, and NMaV (GenBank EU182651) and NCLV (GenBank NC_008266.1) were used as the reference genome and outgroup, respectively. The consensus sequence represents a consensus from all identified NMaV partial sequences. The best-fit model for BIC was TVMe + G4. The numbers shown at the branching points in the phylogenetic tree are called bootstrap values. These values indicate how confident we are in the accuracy of the tree’s structure. To make the defined virus group stand out, we used a differently colored box around it. In addition, the length of each line connecting the branches in the tree tells us about the genetic distance between the viruses. A more extended branch means they are more genetically different, while a shorter branch means they are closely related.

Only one NCLV genome isolate, Zhangzhou, which belongs to the genus *Carlavirus* and encodes six viral proteins, has been reported. Our study obtained 18 NCLV genomes from four yellow and 14 white tepal samples. The phylogenetic tree revealed two distinct genetic groups of NCLV: group A, comprising two isolates (Zhangzhou and Y2R2-1), and group B, consisting of three isolates from yellow tepal samples and 14 isolates from white tepal samples (Figure 4A). Interestingly, different NCLV isolates, such as W3R2, W4R1, and W5R1, were identified in some samples. Specifically, 14 NCLV genomes from white tepal samples showed high similarity and low genetic diversity within the same clade. According to the phylogenetic tree, all 14 NCLV genomes from the white tepal samples originated from two NCLV isolates (Y3R1-1 and Y3R2-1) obtained from the yellow tepal samples.

The complete genome sequence of NMaV is currently unavailable, and only a partial sequence encoding the replicase domain has been reported. In the present study, we obtained many partial sequences encompassing the NMaV replicase domain. However, we did not identify any complete genome sequences or sequences covering other open reading frames (ORFs) of NMaV. From the obtained partial sequences, we selected 16 NMaV sequences with sizes exceeding 900 base pairs (Table S1). A consensus sequence was also generated from all the NMaV partial sequences (Table S1). Remarkably, all NMaV sequences were derived from yellow tepal samples, and multiple variants were identified within the same sample, such as five isolates from Y3R2. Phylogenetic analysis classified the NMaV sequences into three distinct groups: group A, comprising two isolates; group B, comprising six isolates; and group C, comprising ten isolates (Figure 4B).

**Figure 5:**
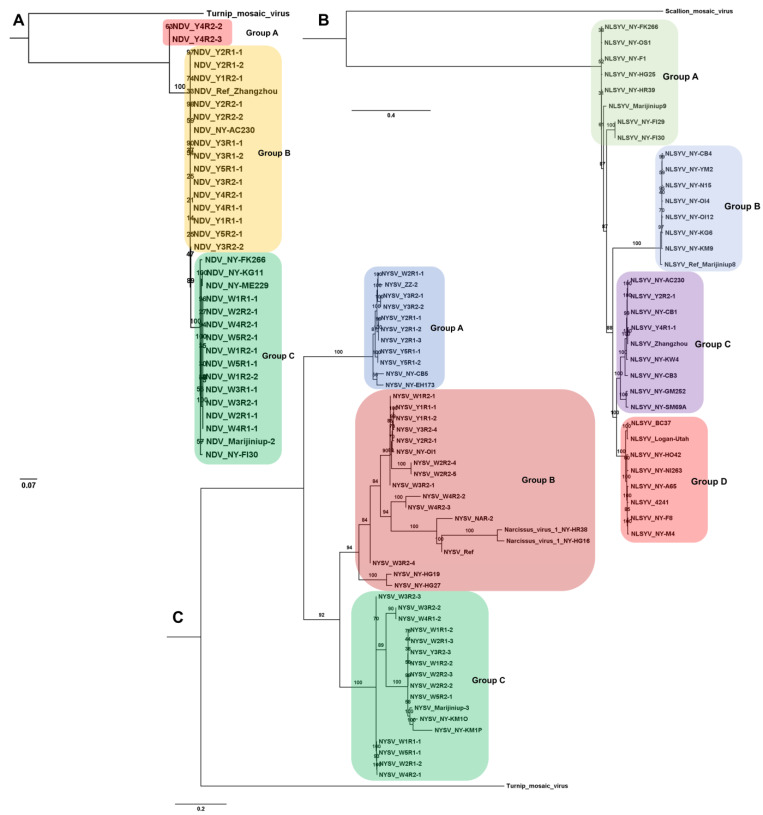
Phylogenetic analysis of NDV, NLSYV, and NYSV isolates. (**A**) The ORFs of 34 NDV isolates were aligned and used for constructing the phylogenetic tree using the Maximum Likelihood method with 1000 bootstrap replicates. NDV (GenBank NC_008824.1) and turnip mosaic virus (GenBank NC_002509.2) was utilized as the reference genome and the outgroup, respectively. The best-fit model based on the BIC was TIM3 + F + G4. (**B**) The ORFs of 33 NLSYV isolates were aligned and used to construct the phylogenetic tree using the Maximum Likelihood method with 1000 bootstrap replicates. NLSYV (GenBank NC_023628.1) and scallion mosaic virus (GenBank NC_003399.1) was used as the reference genome and the outgroup, respectively. The best-fit model based on the BIC was GTR + F + I + G4. (**C**) The whole ORFs of 46 NYSV isolates were aligned and used for constructing the phylogenetic tree using the Maximum Likelihood method with 1000 bootstrap replicates. NYSV (GenBank NC_011541.1) and turnip mosaic virus (GenBank NC_002509.2) was employed as the reference genome and the outgroup, respectively. The best-fit model based on the BIC was GTR + F + I + G4. The numbers shown at the branching points in the phylogenetic tree are called bootstrap values. These values indicate how confident we are in the accuracy of the tree’s structure. To make the defined virus group stand out, we used a differently colored box around it. In addition, the length of each line connecting the branches in the tree tells us about the genetic distance between the viruses. A longer branch means they are more genetically different, while a shorter branch means they are closely related.

We obtained 27 NDV genome sequences, including 16 isolates from yellow tepal samples and 11 isolates from white tepal samples (Table S1). Currently, only seven NDV genome sequences are available in public databases. Phylogenetic analysis revealed three distinct groups of NDV isolates: group A (2 isolates), group B (16 isolates), and group C (16 isolates) (Figure 5A). It appears that the NDV isolates in group C originated from the NDV isolates in group B, derived from the NDV isolates in group A. NDV isolates within the same group exhibited low genetic variability. Interestingly, all 11 NDV isolates belonged to group C, including one isolate (NY-FK266) from *Narcissus jonquilla* in Japan, three isolates from *Narcissus tazetta* in Japan, and one isolate (Marijiniup2) from *Narcissus* species in Australia. Group B included two known NDV isolates from Japan (NY-AC230) and China (Zhangzhou).

The 33 NLSYV isolates, including the two isolates from this study, were categorized into four groups: group A (eight isolates), group B (eight isolates), group C (nine isolates), and group D (eight isolates) (Figure 5B). In this study, two isolates (Y2R2-1 and Y4R1-1) belonged to group C, including six isolates from Japan and one from China (Zhangzhou). Based on phylogenetic analysis, the NLSYV isolates in group A were the ancestors of the isolates in groups B, C, and D. Moreover, the seven NLSYV isolates from Japan and one isolate from China (Marijiniup8) in group B represented more recent evolutionary lineages, according to the phylogenetic tree.

Forty six NYSV isolates were obtained, comprising twenty one from white tepal samples and eleven from yellow tepal samples (Figure 5C). The 46 NYSV isolates were broadly divided into three groups: A (11 isolates), B (18 isolates), and C (17 isolates). The 11 isolates in group A exhibited high sequence similarity. In contrast, 18 isolates in group B and 17 in group C showed high sequence diversity. Group A consisted primarily of seven NYSV isolates from yellow tepal samples and one isolate (W2R1-1) from a white tepal sample. Several variant genomes were identified, including two samples from Y3R2 and Y5R1 and three variants from Y2R1. The isolates in group B exhibited significant sequence diversity, and two isolates of narcissus virus 1 grouped with the NYSV reference genome, indicating their membership in the NYSV. Group B included seven isolates from white tepal samples and four from yellow tepal samples. Interestingly, group C was predominantly composed of white tepal samples (13 isolates) and showed high sequence divergence. It is worth noting that some variants displayed high sequence divergence despite being identified from the same sample, such as W2R1-1 in group A, W2R1-2 in group C, W2R2-4 and W2R2-5 in group B, and W2R2-2 and W2R2-3 in group D.

### 3.6. Genetic Diversity Analysis of NCLV, NDV, NLSYV, and NYSV Isolates

We conducted a comprehensive analysis of the genetic diversity of four viruses (Table 4). The viral populations varied in size, with NYSV having the largest population (46 isolates) and NCLV having the smallest (19 isolates). Among the four viruses, NDV had the highest number of sites (9542 sites), whereas NCLV had the fewest (8513 sites). Notably, NYSV displayed the highest number of segregation sites (4520 sites), indicating greater genetic diversity, whereas NDV had the lowest number of segregation sites (1488 sites). NYSV also exhibited the highest effective number of alleles (6826), suggesting a larger gene pool, whereas NDV had the lowest (1548). The number of haplotypes ranged from 15 (NCLV) to 45 (NYSV), with NLSYV demonstrating the highest haplotype diversity (1) and NCLV the lowest (0.965). Nucleotide diversity was the highest in NYSV (0.20667) and lowest in NDV (0.03409). Additionally, NYSV displayed the highest θw value of 0.1682, indicating the highest estimated population mutation rate (0.1682 per site) among the four viruses. NLSYV exhibited the highest expected heterozygosity (33), indicating substantial genetic diversity. It also demonstrated the highest values for diversity (Hd), nucleotide diversity (Pi), and Watterson’s theta (θw) among the four viruses.

### 3.7. Recombination Analysis of NCLV, NDV, NLSYV, and NYSV

Next, we conducted recombination analyses of the four viruses (Table S2). Our findings revealed a low number of recombination events for the NDV (four events), including five MDV recombinants: Y4R2-2, Y4R2-3, Y2R1-2, Y2R1-1, and Y3R2-2. For NLSYV, we identified four recombination events involving ten isolates: NY-CB1, Y4R1-1, Marijiniup9, NLSYV NY-F1, NY-OS1, NY-HG25, NY-HR39, NY-FK266, and Marijiniup9. For NCLV, eight recombination events were observed, including five recombinants: Y1R1-1, Y2R2-1, Zhangzhou, Y3R2-1, and Y3R1-1. The highest number of recombination events (25) was detected in NYSV, with 24 recombinants identified among the 21 isolates in our study.

**Figure 6:**
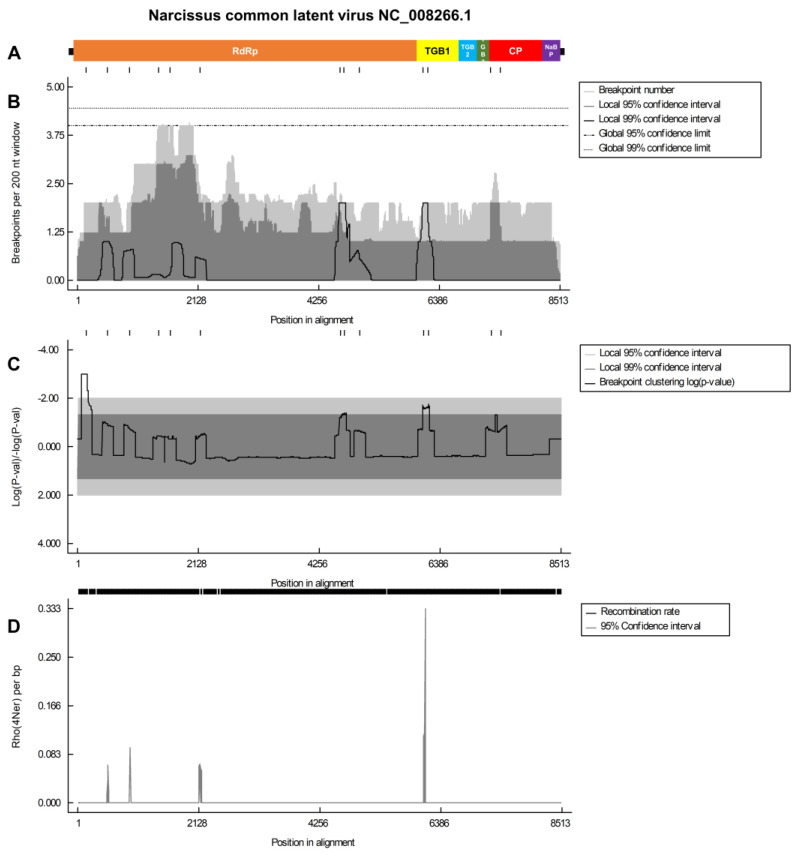
Recombination rate plot and breakpoint distribution analysis of the NCLV genome. (**A**) The organization of the NCLV genome. This depiction focuses on specific individual protein regions within the genome. To understand their positions and organization within the genome, different protein regions are highlighted. (**B**) The distribution of breakpoints in the NCLV genome. Breakpoints are points where genetic recombination occurs, and this plot shows where these recombination events are distributed along the genome. (**C**) The corresponding statistical significance of the breakpoints shown in panel B is depicted. Log-converted *p*-values represent the statistical significance. This helps to assess the reliability and significance of the breakpoints observed in the genome. (**D**) The identification of significant breakpoint clusters in the NCLV genome. We used two permutation tests, one with a 95% confidence level and the other with a 99% confidence level, to identify locally and globally significant clusters of breakpoints. These tests help determine if the observed clusters are statistically significant and not just due to random chance.

**Figure 7:**
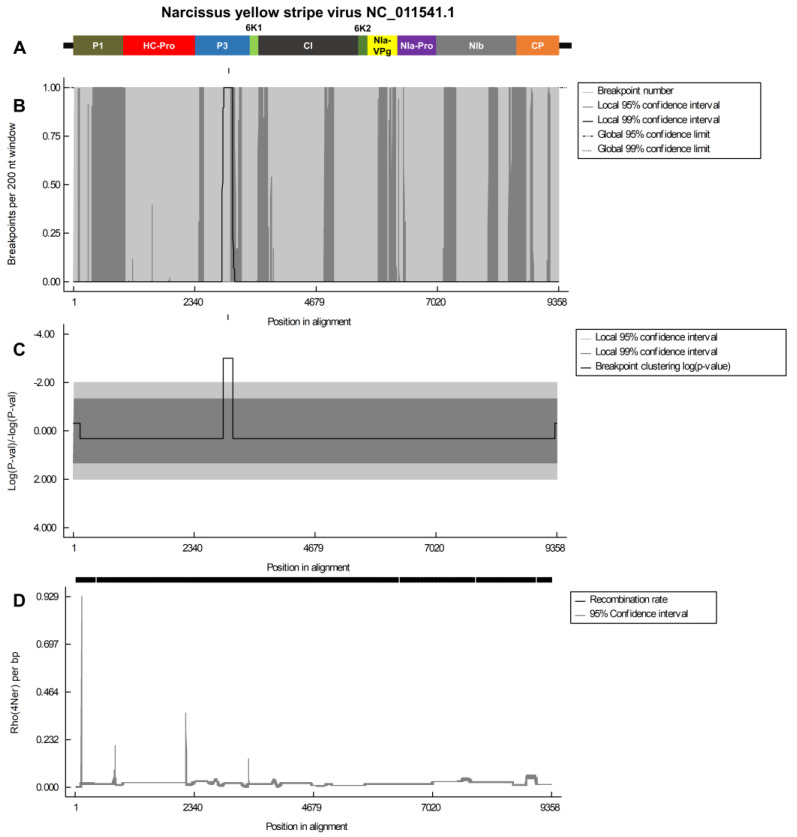
Recombination rate plot and breakpoint distribution analysis of the NYSV genome. (**A**) The organization of the NYSV genome. This depiction focuses on specific individual protein regions within the genome. Different protein regions are highlighted to understand their positions and organization within the genome clearly. (**B**) The distribution of breakpoints in the NYSV genome. Breakpoints are points where genetic recombination occurs, and this plot shows where these recombination events are distributed along the genome. (**C**) The corresponding statistical significance of the breakpoints shown in panel B is depicted. Log-converted *p*-values represent the statistical significance. This helps to assess the reliability and significance of the breakpoints observed in the genome. (**D**) The identification of significant breakpoint clusters in the NYSV genome. We used two permutation tests, one with a 95% confidence level and the other with a 99% confidence level, to identify locally and globally significant clusters of breakpoints. These tests help determine if the observed clusters are statistically significant and not just due to random chance.

Subsequently, we analyzed the recombination breakpoints to identify the specific genomic regions in which these events occurred. Of the four viruses, the NCLV genome exhibited the highest breakpoints (Figure 6). Notably, Rho per bp analysis revealed three peaks in the RdRp region and a single highly elevated peak in the TGB1 region of NCLV, indicating potential recombination events (Figure 6). In NYSV, multiple breakpoints were identified along the viral genome, including regions P1, P3, C1, NIa-VPg, NIb, and CP (Figure 7). Statistical analysis strongly supported the presence of breakpoints in the P3 region. Among the various peaks observed in the Rho per bp graph, the peak in the P1 region exhibited the highest Rho per bp (Figure 7).

The NDV genome displayed few breakpoints, with only the P3 region exhibiting a high breakpoint value (Figure S1). Moreover, Rho per base pair analysis revealed no peaks, suggesting no recombination events occurred in the examined NDV genomes. For NLSYV, both the P1 and CP regions showed higher breakpoints (Figure S2). Analysis of Rho per base pair revealed elevated peaks in the P1 and P3 regions of NLSYV, indicating potential recombination events.

## 4. Discussion

In this study, we investigated the viromes in Chinese narcissus flowers using transcriptome data from 20 samples collected at various stages of flower development. Through a series of data processing steps, we identified seven distinct virus species in the narcissus transcriptome: NCLV, NMaV, NSV, CMV, NDV, NLSYV, and NYSV. Similarly, previous studies have revealed coinfection with multiple viruses that infect *Narcissus* species. For example, in China, three viruses (NCLV, NYSV, and NLSYV) have been identified in *Narcissus tazetta* using reverse transcriptase-polymerase chain reaction (RT-PCR) with conserved carlavirus and potyvirus primers [3]. In New Zealand, enzyme-linked immunosorbent assays (ELISA) and mechanical transmission tests have detected five viruses (CMV, NLV, narcissus mosaic virus [NMV], narcissus tip necrosis virus, and NYSV) from diverse *Narcissus* species [10]. In Australia, two viruses (NLSYV and vallota speciosa virus) were identified in domestic and wild *Narcissus* species [26]. Furthermore, in Japan, RT-PCR has detected two potyviruses (NLSYV and NDV) in the narcissus [4]. Our study identified the highest number of viruses infecting Chinese narcissus plants using high-throughput sequencing (HTS) techniques and many plant samples. Based on previous results, plant samples and geographical regions are important factors in determining virus populations.

HTS and bioinformatics analyses enabled us to quantify the viral load in each sample. In particular, the analysis of viral reads and transcripts per million (TPM) provides insights into the abundance of each virus in samples [12,13]. NYSV was most abundant among the identified viruses, followed by NDV, NCLV, and NMaV. Interestingly, the proportion of viral reads varied between the white and yellow tepal plants. White tepal plants showed an increasing proportion of viral reads with the flower development stage, whereas the difference in viral proportion among yellow tepal plants was relatively small. Based on these results, we hypothesized that the viral load increases as the plant grows; however, this phenomenon highly depends on the plant cultivar or species. Further examination of the composition of the identified viruses in each sample revealed that NDV was the most abundant virus in most of the white and yellow tepal samples. NYSV was also prominent in yellow tepal samples. Potyviruses, including NDV, NLSYV, and NYSV, were the major viruses infecting Chinese narcissus in this study. This is the first study to report viral populations in the diverse flower tissues of higher plants. Therefore, our results provide information about the dominant virus in each sample, which cannot be readily determined using other virus detection techniques, such as RT-PCR and ELISA.

Moreover, our study demonstrated that flower tissue is suitable for virus detection in narcissus plants because of the high viral load, regardless of the developmental stage. The viral abundance in narcissus flower tissues during the examined developmental stages was significantly higher than in other plant species, such as sweet potato [27] and lily [28]. Selecting the appropriate plant tissue is crucial for virus detection, as extracting high-quality total RNA from plant leaves enriched with polysaccharides and polyphenols can be challenging [29]. Therefore, we strongly suggest that flower tissue from *Narcissus* spp. is an excellent material for virus detection.

*De novo* transcriptome assembly enabled us to obtain the viral contigs for further analysis. The obtained contigs varied in size, with some contigs associated with NMaV being less than 1000 bp. We identified the putative viral genome sequences for NYSV, NLSYV, and NDV by applying a size cutoff. Obtaining a large number of viral genomes in a virome study facilitates studies related to viral genome-associated aspects, such as the phylogenetics, diversity, and evolutionary rates of the identified viruses [4,11,30]. Remarkably, 80 viral genome sequences encompassing open reading frames (ORFs) were obtained within a single study. Interestingly, we found that carlaviruses, such as NCLV and the three potyviruses, had poly(A) tails that could be detected by RNA sequencing generated from cDNA using oligo-d(T) [31]. Moreover, based on our experience, longer sequence lengths achieved through high-throughput sequencing (HTS) (here, 150 bp was used instead of 100 bp) are crucial for obtaining complete viral genome sequences. For NMaV, only partial sequences were obtained, indicating that an alternative approach should be considered for obtaining the complete NMaV genome soon.

Phylogenetic analyses of the identified viral genomes provided insights into their genetic relationships. The NCLV, NMaV, NDV, NLSYV, and NYSV have distinct genetic groups. Multiple NCLV isolates were identified in the same sample, indicating the presence of genetic variants. The NMaV sequences were classified into three distinct groups, with multiple variants identified within the samples. The NDV isolates exhibited three distinct groups, where group C isolates originated from group B and were derived from group A. NLSYV isolates were categorized into four groups, with group A isolates being the ancestors of groups B, C, and D. NYSV isolates were broadly divided into three groups, with groups B and C exhibiting high sequence diversity.

One of the most intriguing findings of this study was the phylogenetic analysis of both NDV and NCLV, which showed that the two viruses found in yellow tepal plants were ancestors of the viruses present in white tepal plants. Previous studies suggested that yellow tepal plants mutated from the white tepal plant known as the ‘Jinzhan Yintai’ cultivar [19]; however, no biological evidence supported this claim. Based on our results, it is likely that white tepal plants originated from yellow tepal plants. Furthermore, many viruses identified in yellow tepal plants exhibited more significant divergence than those in white tepal plants. This suggests that yellow tepal plants may represent wild populations in diverse regions, whereas white tepal plants are domesticated narcissus plants clonally propagated on a large scale and commonly found in many places. However, further experiments are required to confirm this hypothesis.

Analysis of genetic diversity among the NCLV, NDV, NLSYV, and NYSV isolates provided insights into the variation within these viral populations. Variants were identified even within samples, indicating the presence of diverse viral genomes within the same host. These results suggest that the genetic diversity of the identified viruses was significantly higher in yellow tepal plants than in white tepal plants, indicating host-dependent viral mutations and evolution [32].

Overall, this study revealed the presence of multiple viruses in Chinese narcissus flowers and shed light on their abundance, composition, genetic diversity, and relationships. These findings enhance our understanding of the narcissus flower virome and emphasize the prevalence of potyviruses in this plant species. Further research is warranted to investigate the impact of these viruses on the health and productivity of narcissus.

## 5. Conclusions

In conclusion, this study explored the viromes in Chinese narcissus flowers using transcriptome data from 20 samples collected at various stages of flower development. Seven distinct viral species have been identified: NCLV, NMaV, NSV, CMV, NDV, NLSYV, and NYSV. This study identified the highest number of viruses infecting *Narcissus tazetta* plants to date, using high-throughput sequencing techniques and a large number of plant samples. The results revealed that the viral loads varied between white and yellow tepal plants, with white tepal plants showing an increasing proportion of viral reads during flower development. NDV was the most abundant virus in most samples, followed by NYSV, NCLV, and NMaV. Potyviruses, including NDV, NLSYV, and NYSV, are the major viruses infecting Chinese daffodils.

Furthermore, this study demonstrated that flower tissue is suitable for virus detection in narcissus plants because of its high viral load, regardless of the developmental stage. Flower tissues exhibited significantly higher viral abundance than other plant species. The use of high-throughput sequencing and bioinformatic analysis has allowed the identification of viral contigs and putative viral genome sequences. Phylogenetic analysis provided insights into the genetic relationships between the identified viruses, revealing the distinct genetic groups of NCLV, NMaV, NDV, NLSYV, and NYSV. Notably, phylogenetic analyses suggested that the viruses found in yellow tepal plants were the ancestors of white tepal plants, challenging previous assumptions regarding the origin of different narcissus cultivars.

This study contributes to our understanding of the virome in Chinese narcissus flowers by highlighting the prevalence of potyviruses and providing information on the dominant viruses in each sample. These findings also emphasize the importance of selecting appropriate plant tissues for virus detection and the impact of host-dependent viral mutations and evolution on genetic diversity. Further research is needed to investigate the implications of these viruses on the health and productivity of narcissus.

## Figures and Tables

**Table 1:** Sample information of Chinese narcissus flower virome study.

Sample Name	Library Name	Accession No.	Cultivar	Developmental Stage	Tepal Color
W1R1	T01	SRR19993659	Jinzhan Yintai	S1	White
W1R2	T02	SRR19993658	Jinzhan Yintai	S1	White
W2R1	T03	SRR19993647	Jinzhan Yintai	S2	White
W2R2	T04	SRR19993646	Jinzhan Yintai	S2	White
W3R1	T05	SRR19993645	Jinzhan Yintai	S3	White
W3R2	T06	SRR19993644	Jinzhan Yintai	S3	White
W4R1	T07	SRR19993643	Jinzhan Yintai	S4	White
W4R2	T08	SRR19993642	Jinzhan Yintai	S4	White
W5R1	T09	SRR19993641	Jinzhan Yintai	S5	White
W5R2	T10	SRR19993640	Jinzhan Yintai	S5	White
Y1R1	T11	SRR19993657	Yellow mutant	S1	Yellow
Y1R2	T12	SRR19993656	Yellow mutant	S1	Yellow
Y2R1	T13	SRR19993655	Yellow mutant	S2	Yellow
Y2R2	T14	SRR19993654	Yellow mutant	S2	Yellow
Y3R1	T15	SRR19993653	Yellow mutant	S3	Yellow
Y3R2	T16	SRR19993652	Yellow mutant	S3	Yellow
Y4R1	T17	SRR19993651	Yellow mutant	S4	Yellow
Y4R2	T18	SRR19993650	Yellow mutant	S4	Yellow
Y5R1	T19	SRR19993649	Yellow mutant	S5	Yellow
Y5R2	T20	SRR19993648	Yellow mutant	S5	Yellow

**Table 2:** Identification of viruses in 20 Chinese narcissus flower transcriptomes.

Accession No.	Virus Name	Abbreviation	Genus	No. of Contigs	No. of Reads
NC_008266.1	Narcissus common latent virus	NCLV	*Carlavirus*	2913	2,292,293
EU182651.1	Narcissus mottling-associated virus	NMaV	*Carlavirus*	463	1,974,398
NC_008552.1	Narcissus symptomless virus	NSV	*Carlavirus*	30	48,321
NC_002034.1	Cucumber mosaic virus	CMV_RNA1	*Cucumovirus*	3	14
NC_002035.1	Cucumber mosaic virus	CMV_RNA2	*Cucumovirus*	1	20
NC_001440.1	Cucumber mosaic virus	CMV_RNA3	*Cucumovirus*	2	101
NC_008824.1	Narcissus degeneration virus	NDV	*Potyvirus*	266	5,938,113
NC_023628.1	Narcissus late season yellows virus	NLSYV	*Potyvirus*	360	657,664
NC_011541.1	Narcissus yellow stripe virus	NYSV	*Potyvirus*	1855	7,461,407

**Table 3:** Size distribution of identified viral contigs associated with five viruses.

Contig Size (bp)	NCLV	NYSV	NMaV	NLSYV	NDV
~1000	2665	1599	457	243	228
1001~2000	114	61	6	33	5
2001~3000	34	39	0	33	0
3001~4000	31	46	0	16	1
4001~5000	11	26	0	6	0
5001~6000	17	7	0	4	0
6001~7000	10	22	0	14	2
7001~8000	7	7	0	6	0
8001~9000	22	7	0	0	3
9001~10,000	2	30	0	3	26
10,001~	0	11	0	2	1

**Table 4:** Analyses of genetic diversity of four viruses infecting Chinese narcissus.

Virus	Number of Isolates	Sites	S	Eta	H	Hd	Pi	θw
NCLV	19	8513	2447	2725	15	0.965	0.06205	0.09338
NDV	34	9542	1488	1548	29	0.984	0.03409	0.03968
NLSYV	33	9297	3018	3438	33	1	0.11162	0.09112
NYSV	46	9234	4520	6826	45	0.999	0.20667	0.1682

Sites, total number of sites; S, the number of segregating sites; Eta, total number of mutations; H, number of haplotypes; Hd, haplotype diversity; Pi, nucleotide diversity π (Pi); θw, Watterson’s estimator of θ.

## Data Availability

The raw datasets (SRR19993640-SRR19993659) associated with the accession number PRJNA855612 are available from the Sequence Read Archive (SRA) database at the National Center for Biotechnology Information (NCBI).

## Supplementary Materials

The following supporting information can be downloaded from: https://www.mdpi.com/article/10.3390/biology12081094/s1, Table S1: Complete genome sequences of 19 NCLV isolates, 27 NDV isolates, 2 NLSYV isolates, and 33 NYSV isolates, along with 17 partial sequences of NMaV from this study. Table S2: Recombination results identified using the RDP5 program for NCLV, NDV, NLSYV, and NYSV. Figure S1: Recombination rate plot and breakpoint distribution analysis of the NDV genome. Figure S2: Recombination rate plot and breakpoint distribution analysis of the NLSYV genome. Supplementary methods: The commands (codes) for the programs used in the Section 2.
