# Supplementary material for: In Silico Virome Analysis of Chinese Narcissus Transcriptomes Reveals Diverse Virus Species and Genetic Diversity at Different Flower Development Stages
Source: Biology (Basel). 2023 Aug 5;12(8):1094. doi: 10.3390/biology12081094 (PMC10452245; doi:10.3390/biology12081094)
Supplement: Supplementary file 1 [file biology-12-01094-s001.zip › Supplementary methods.pdf]

The commands (codes) for the programs used in the Materials and Methods section are as follows:

- Obtain raw datasets from the SRA database:

```
prefetch SRR19993640-SRR19993659
```

- Convert SRA data to FASTQ format using the SRA-Toolkit:

```
fastq-dump SRR19993640-SRR19993659
```

- Quality control, trimming, and filtering of low-quality reads using BBDuk:

```
bbduk.sh in=raw_reads.fastq out=high_quality_reads.fastq qtrim=rl trimq=20
```

- *De novo* transcriptome assembly using Trinity:

```
Trinity --seqType fq --left high_quality_reads_R1.fastq --right high_quality_reads_R2.fastq --CPU 8 --output transcripts_assembly
```

- BLASTX search against the viral protein database:

```
blastx -query contigs.fasta -db viral_protein_db -evalue 1E-10 -out results_blastx_viral.txt
```

- Further BLASTX search against the non-redundant protein database:

```
blastx -query viral_contigs.fasta -db nr_db -evalue 1E-5 -out viral_contigs_identification.txt
```

- Align raw sequence reads against reference viral genomes using BWA aligner:

```
bwa mem -t 8 reference_viral_genomes.fasta raw_reads.fastq > alignment.sam
```

- Calculate coverage, viral reads, and transcripts per million (TPM) using eXpress:

```
express --output-dir expression_output alignment.sam
```

- Annotate viral genome using ORFfinder:

```
orffinder -sequence viral_contigs.fasta -outfmt gff -minsize 100 -out viral_genome_annotation.gff
```

- Perform a BLASTX search against a non-redundant protein database:

```
blastx -query predicted_orfs.fasta -db nr_db -evalue 1E-5 -out viral_genomic_features.txt
```

- Align viral sequences using MAFFT:

```
mafft --auto viral_genome_sequences.fasta > aligned_sequences.fasta
```

- Determine the best-fitting model for phylogenetic tree construction:

```
iqtree -s aligned_sequences.fasta -m MFP
```

- Construct phylogenetic tree using IQ-TREE with 1000 bootstrap replicates:

```
iqtree -s aligned_sequences.fasta -m MODEL -bb 1000
```

- Use aligned sequences for genetic diversity analysis using DnaSP6:

```
dnasp -sequence aligned_sequences.fasta
```

- Perform recombination analysis using RDP version 5.23:

```
rdp5 -a -c -f 4 -p 0.05 -s output_recombination_results input_sequence.fasta
```
