# Supplementary figures and images for: In Silico Virome Analysis of Chinese Narcissus Transcriptomes Reveals Diverse Virus Species and Genetic Diversity at Different Flower Development Stages
Source: Biology (Basel). 2023 Aug 5;12(8):1094. doi: 10.3390/biology12081094 (PMC10452245; doi:10.3390/biology12081094)

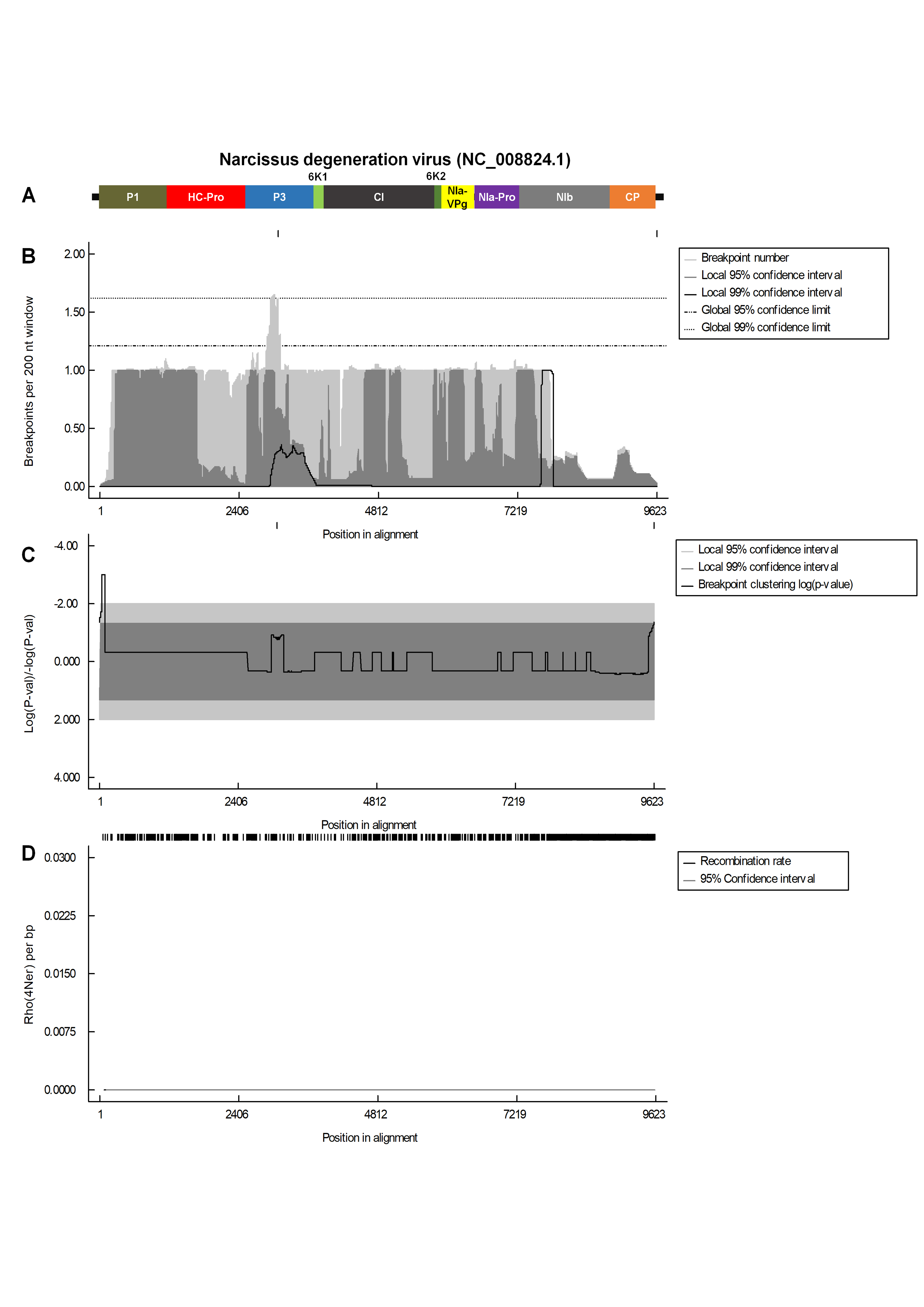

Supplement: Supplementary file 1 [file biology-12-01094-s001.zip › Figure S1.jpg]

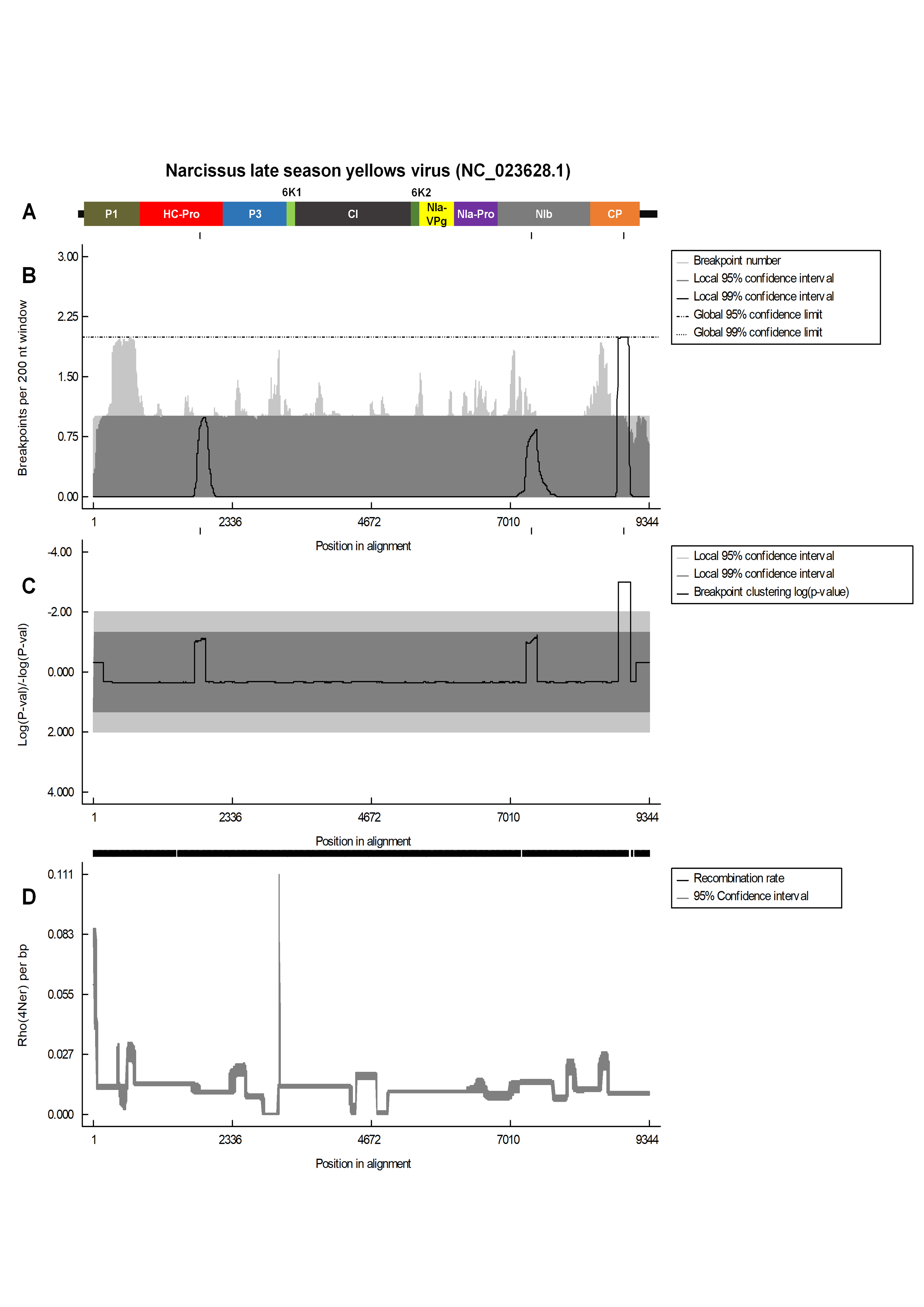

Supplement: Supplementary file 1 [file biology-12-01094-s001.zip › Figure S2.jpg]
